# RNA sequencing analysis of sexual dimorphism in Japanese quail

**DOI:** 10.3389/fvets.2024.1441021

**Published:** 2024-07-22

**Authors:** Sinwoo Park, Jaeryeong Kim, Jinbaek Lee, Sungyoon Jung, Seung Pil Pack, Jin Hyup Lee, Kyungheon Yoon, Seung Je Woo, Jae Yong Han, Minseok Seo

**Affiliations:** ^1^Department of Computer and Information Science, Korea University, Sejong-si, Republic of Korea; ^2^Department of Biotechnology and Bioinformatics, Korea University, Sejong-si, Republic of Korea; ^3^Department of Food and Biotechnology, Korea University, Sejong-si, Republic of Korea; ^4^Division of Genome Science, Department of Precision Medicine, National Institue of Health, Cheongju-si, Republic of Korea; ^5^Department of Agricultural Biotechnology and Research Institute of Agriculture and Life Sciences, Seoul National University, Seoul, Republic of Korea

**Keywords:** RNA-sequencing, Japanese quail, sexual dimorphism, Z chromosome, avian

## Abstract

**Introduction:**

Japanese quail are of significant economic value, providing protein nutrition to humans through their reproductive activity; however, sexual dimorphism in this species remains relatively unexplored compared with other model species.

**Method:**

A total of 114 RNA sequencing datasets (18 and 96 samples for quail and chicken, respectively) were collected from existing studies to gain a comprehensive understanding of sexual dimorphism in quail. Cross-species integrated analyses were performed with transcriptome data from evolutionarily close chickens to identify sex-biased genes in the embryonic, adult brain, and gonadal tissues.

**Results:**

Our findings indicate that the expression patterns of genes involved in sex-determination mechanisms during embryonic development, as well as those of most sex-biased genes in the adult brain and gonads, are identical between quails and chickens. Similar to most birds with a ZW sex determination system, quails lacked global dosage compensation for the Z chromosome, resulting in directional outcomes that supported the hypothesis that sex is determined by the individual dosage of Z-chromosomal genes, including long non-coding RNAs located in the male hypermethylated region. Furthermore, genes, such as *WNT4* and *VIP,* reversed their sex-biased patterns at different points in embryonic development and/or in different adult tissues, suggesting a potential hurdle in breeding and transgenic experiments involving avian sex-related traits.

**Discussion:**

The findings of this study are expected to enhance our understanding of sexual dimorphism in birds and subsequently facilitate insights into the field of breeding and transgenesis of sex-related traits that economically benefit humans.

## Introduction

Japanese quail (*Coturnix japonica*), a representative poultry, takes approximately five to six weeks to reach sexual maturity, compared to the six month period for chickens (1). Additionally, the interval between generations is relatively short. Raising quails is low cost and their protein-rich eggs and meat have important economic value (2, 3). Poultry, including quail and chickens, are used for protein, an essential nutrient for humans. Most males that cannot produce eggs are slaughtered during breeding. More than 600 million male chicks are annually slaughtered worldwide, causing economic and ethical problems (4, 5). Therefore, an accurate understanding of the sexual dimorphism of quail is important for the efficiency of breeding as an economic animal as well as providing solutions to bioethical issues.

In birds with the ZW sex chromosome system (ZZ for male and ZW for female), a clear sex determination mechanism has not been identified to date, and there are two main hypotheses for sex determination in birds: (1) W-chromosomal genes may determine female (6, 7), and (2) sex may be determined according to the dosage of Z-chromosomal genes (8). The avian W chromosome corresponds to the Y chromosome of the mammalian XY sex determination system. In mammals, the expression of sex-determining region Y (SRY) influences sex determination; however, in birds, a gene that plays a similar role has not yet been identified (9, 10). On the other hand, the results of numerous studies conducted to date in chickens, a model organism for birds, give weight to the hypothesis that sex can be determined by the dosage of Z-chromosomal genes (11, 12). In this regard, dosage compensation is an important mechanism in sexual dimorphism, in which the expression of genes located on sex chromosomes is suppressed or overexpressed to balance the expression levels of sex chromosomal genes (13, 14). In mammals, the expression levels of X-chromosomal genes are kept balanced between homogametic (XX) and heterogametic (XY) through X-chromosomal inactivation mechanism is triggered by *XIST*, a X-chromosomal long non-coding RNA (lncRNA) (15). However, the current consensus is that in birds, the master gene responsible for global transcriptional inactivation of Z-chromosomal genes is unknown, and there is no compensation of the entire Z-chromosome (16, 17). Another axis of research on sexual dimorphism in birds involves male-specific epigenetic phenomena. There is a male hypermethylated (MHM) region on the Z chromosome, where high levels of methylation inhibit gene expression in male chickens (18, 19). In the MHM region of females, the central histone H4 protein is acetylated, increasing gene expression, whereas this phenomenon does not occur in males (19, 20). Therefore, sexual dimorphism studies on chicken, such as dosage compensation and those on the MHM region, have been conducted; however, in the case of quails, which are experimental non-model species, research remains limited.

Research to identify biomarkers related to sex-determination mechanisms and sex differences in birds has been primarily conducted based on transcriptome data. Most of these studies were conducted in chickens, and several sexually dimorphic genes have been identified. *DMRT1* plays an important role in male reproductive organs formation (21, 22). Although an incompletely feminized subject incapable of reproductive activity was established, a *DMRT1* disruption experiment on early chicken embryos demonstrated that it is possible to change the sex of a chicken from male to female (23). Additionally, *SOX9* was established as a male sex regulator that functionally cooperates with *DMRT1* (24). Furthermore, various sexual dimorphism-related genes, such as *WNT4*, *CYP19A1*, and *FOXL2*, which affect the development of female reproductive organs and reproductive functions, have been identified. Data on other bird species remain unknown because of the low accessibility of sequencing technology (25) and the incomplete reference genome and gene annotation. Although new reference genomes for several bird species have been established since the launch of The Bird 10,000 Genomes (B10K) Project (26), they have not yet reached the level of model organisms. Among the numerous bird species that currently exist worldwide, there are only 52 species for which reference genomes and gene annotations have been established in the Ensembl database (27). Due to the absence of a quail reference genome, research on sexual dimorphism was conducted based on reference-free methods, but the precision of the research was limited (28, 29). The official reference genome (*Coturnix_japonica_2.0*) and gene annotation for Japanese quail were only established in 2016, therefore, transcriptome data on sexual dimorphism are still lacking compared to those for chickens. Moreover, a second practical difficulty in the limited transcriptome studies of sex determination mechanisms in birds arises from the nature of the early embryonic development process in birds. In birds, the initial stages of embryonic maturation, before sex determination, occur inside the eggshell within the parent uterus; therefore, the development of noninvasive methods to obtain intrauterine embryos must take precedence. In chickens, as these technical limitations have been resolved, not only early embryo studies (30, 31) but also diverse transcriptome studies at the single-cell level have been successfully conducted (32, 33). However, research on sexual dimorphism in quails is still in its infancy (34).

In response to the necessity for research on sexual dimorphism in quail, this study collected publicly available quail RNA-seq data as of May 2024. In addition, we collected RNA-seq data relevant to study of sexual dimorphism in chickens, an evolutionarily close relative of quail, which has been studied at a relatively high level in sexual dimorphism transcriptome studies. Based on the collected data, we conducted an *in-silico* cross-species integrated analysis to explore sexual dimorphism in quail.

## Materials and methods

### RNA-seq data collection

**Figure 1:**
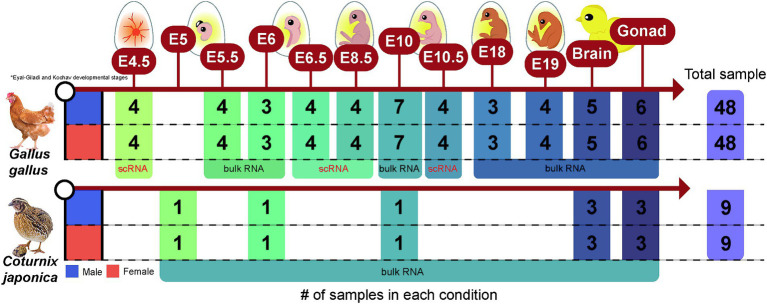
Data structure of a total of 114 RNA-sequencing data collected from quail and chicken. The number of samples of RNA-sequencing data collected for each species in each condition (embryonic stage, brain, or gonad) is presented.

In total, 114 raw RNA-seq data (18 and 96 samples for quail and chicken, respectively) were collected from the Sequence Read Archive (SRA) and DNA Data Bank of Japan databases (Supplementary Table S1). Six bulk RNA-seq datasets generated at E5, E6, and E10, the stages of sex differentiation during embryonic development in quail, were collected from PRJDB7305, and 12 bulk RNA-seq datasets derived from adult brain tissue and gonads in quail were collected from PRJNA272589. We also collected RNA-seq data generated from evolutionarily close chickens to compensate for the relative lack of samples from quail, a non-model avian species, using cross-species integrated analyses. Seventy-four RNA-seq data corresponding to the chicken embryonic developmental stages E4.5–E19 were collected from six independent projects (PRJNA600155, PRJNA766745, PRJNA871063, PRJNA284655, PRJNA867032, and PRJNA631788). Of these, 32 were single-cell RNA-seq (scRNA-seq) data, and the remaining 42 were bulk RNA-seq data. Ten bulk RNA-seq reads from adult brain tissues of chickens were collected from PRJNA551402 and PRJNA656654, and 12 bulk RNA-seq reads from adult gonadal tissues of chickens were collected from PRJNA867032 and PRJNA613236. All RNA-seq data were collected as raw sequencing data using the fastq-dump tool from the SRA toolkit.^1^ In summary, the 114 RNA-seq datasets collected consisted of three main variables: sex (male and female), source (embryonic stage, brain, and gonads), and species (quail and chicken) (Figure 1).

### Preprocessing for RNA-seq data

Bulk RNA-Seq Data (.fastq) were mapped to the corresponding GRCg6a (chicken) and *Coturnix_japonica_2.0* (quail) reference genomes using HISAT2 (v2.2.1) (35), respectively. The resulting sam files were then converted to.bam format using ‘samtools view -Sb -@ 16’ and then sorted using ‘samtools sort -o output.bam -@ 16’ (36). Finally, the quantification of mapped reads was performed using FeatureCounts (v2.0.0) (37) with the ‘featureCounts -p -a XXX.gtf -t exon -g gene_id -T 32’ command. The gene annotation build used Ensembl Gene 111 for each species.

Of the collected data, 32 RNA-seq data points from specific stages of chicken embryonic development were scRNA-seq rather than bulk RNA-seq data. To make this compatible with the rest of the bulk RNA-seq data, pseudo-bulk aggregation was performed based on the raw scRNA-seq data. Raw scRNA-seq data (.fastq.gz) were preprocessed using Cell Ranger (v6.0.0) (38) with the command ‘cellranger count --id = SampleID --fastqs = rawfastq.fastq.gz --sample = $sample --transcriptome = GRCg6a --localcores = 8 --localmem = 32’. A cell-by-gene count matrix (.mtx) per sample was loaded using the readMM() function in the Matrix R package (v1.7.0). Finally, the rowSums() function was used to generate a pseudo-bulk count matrix for each sample.

### Statistical analysis to identify sex-biased genes

edgeR (v.3.40.2) (39) was used to identify sex-biased genes in each species (quail and chicken) and the associated conditions (embryonic stages: E4.5–E19, brain, and gonad). Two-group comparisons between male and female were performed independently for each condition. For all tests, trimmed mean of M-value normalization was applied to correct for differences in library size between samples. Each statistical hypothesis test included at least three biologically replicated samples within the groups, and the sample sizes within the conditions for males and females were always balanced (Figure 1). The *p*-values resulting from the hypothesis tests were adjusted for multiple testing using Benjamini and Hochberg’s false discovery rate (FDR) adjustment method (40), with significance set at FDR-adjusted *p* ≤ 0.05.

### Functional enrichment analysis

Functional enrichment analysis was conducted using the DAVID tool (41) to investigate the functional characteristics of the identified sexually dimorphic genes. As the maximum number of limit genes in the DAVID software was 3,000, we selected the top genes based on log2 fold change (logFC) for enrichment analysis of genes beyond this limit. *C. japonica* was used as the background for the enrichment analysis. *p* ≤ 0.01 was considered as statistically significant for the functional term.

### The classification of sex-biased patterns by tissue specificity

Sex-biased genes were classified based on logFC, with the objective of determining whether the expression patterns of these genes were tissue-specific. The observation of three distinct patterns of sex bias (male-biased, non-significant, female-biased) in each brain and gonadal tissue led to the identification of nine patterns among their combinations. Eight distinct directions were identified for the sex-biased genes, with the exception of those that were not significantly different in either tissue. Detailed criteria for the classification of the eight pattern groups of sex-biased genes and the interpretation of each pattern are presented in Supplementary Table S2.

### Identification of orthologous gene between quail and chicken genomes

For cross-species integrated analysis of genes annotated in two different species, quail and chicken, orthologous genes were identified based on genomic information obtained from the Ensembl biomart (42). Only genes with one-to-one homology and orthologous pairs with a high orthology confidence level were considered as orthologous genes between the two species. Orthologs of lncRNAs between the two species were excluded. Consequently, orthologous genes of sex-biased lncRNAs identified through RNA-seq analysis in quail were further identified using the following process: the BLAST database for GRCg6a was used to perform a homology search against the chicken genome using blastn (43) with the following command: ‘makeblastdb -in GRCg6a.fasta -dbtype nucl -out GRCg6a’; the lncRNA sequences found in quail were listed in the query.fasta file; and a blast search was performed using the command with ‘blastn -query query.fasta -db GRCg6a -out results.txt -outfmt 5′ against the established GRCg6a database. Sequence homology search results were obtained in XML format and visualized using the Kablammo tool (44) when visualization was required. The same process was repeated for the higher version of the reference genome, GRCg7b.

### Quality assessment of reference genome and gene annotation

The NGS applicability index (25) was used to assess the quality of gene annotation using the reference genome. The following genome assembly quality measures were simultaneously considered in the evaluation of the reference genome: AdjN50Contig, AdjN50Scaffold, UngapRate, Uniquely mapped rate, mapping rate, and multi-mapping rate. In addition, the quality metrics of transcript diversity, quantification rate, quantification rate (Abs), and quantification rate (Amb) were simultaneously considered for gene annotation evaluation. To calculate the NGS applicability index, the weight of each metric was set to 1.

## Results

### Reference genome-based approaches can more accurately identify sex-biased genes in quail

Research to identify sex-biased genes based on RNA-seq data targeting Japanese quail species has been conducted since 2014, but there was no publicly available reference genome at the time, analysis was performed using a *de novo* approach (29, 45). However, we presumed that the analysis was performed without the reference genome, which provides relatively stable information, leading to inaccurate results owing to diverse technical problems. To investigate this hypothesis, we reanalyzed the identification of sex-biased genes in the adult reproductive organs and brains of quail based on the *Coturnix_japonica_2.0* reference genome released in March 2016. We compared the ratio of mapped reads using the *de novo* approach performed in 2015 with the reference genome-based method used in this study. An average of 85.202% of all generated reads were successfully mapped to the reference genome, whereas the analysis method using the *de novo* approach mapped an average of only 69.658% of the reads to the assembled transcript (Supplementary Figure S1A). Additionally, the standard deviation of the reference genome-based approach was 0.64, showing stable results compared with the *de novo* approach of 2.92. These results indicated that reference genome-based analysis can yield more stable results, as expected.

Evidence of the stability of the reference genome-based approach was also found in the number of genes whose expression levels were successfully quantified. The number of genes quantified using the reference genome-based method was 19,341, but an existing study identifying sex-biased genes through a *de novo* approach was conducted on only 3,503 assembled transcripts (29). As a result of the dimensionality reduction for 19,341 genes whose expression levels were measured using a more stable reference genome-based approach, we observed notable differences between samples derived from different gonadal tissues (Supplementary Figure S1B). In contrast, relatively small sex differences were observed in samples derived from brain tissue. The pattern of relationships between samples was identical to that obtained using a previously performed *de novo* approach (29).

### Characterization of sex-biased genes in Japanese quail’s brain

**Figure 2:**
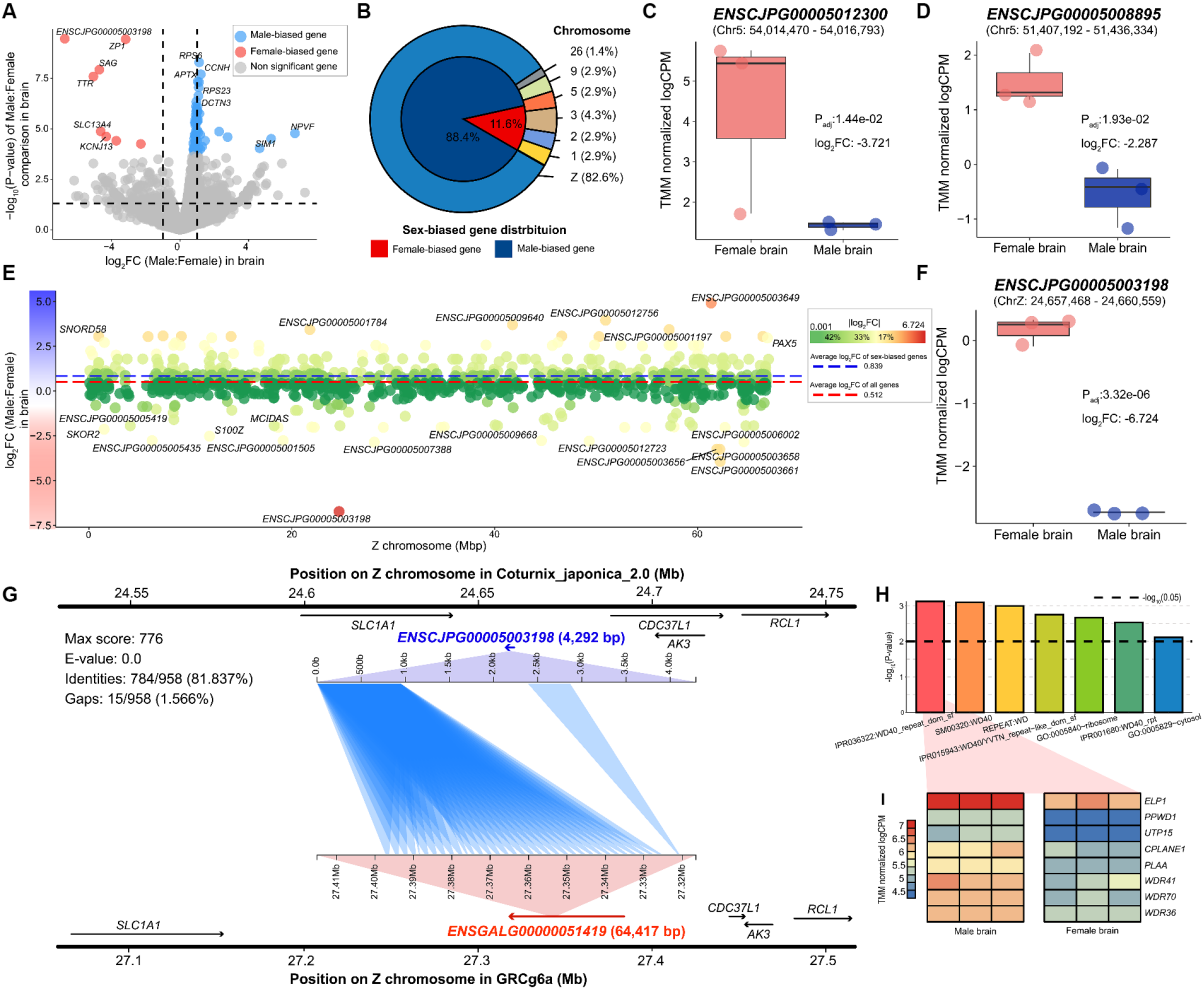
Sex-biased genes identified in quail brain. **(A)** Sex-biased genes significantly discovered in quail brain tissue. The x-axis represents the log_2_ fold-change values between males and females, where a positive value indicates male-biased direction. The y-axis shows the -log_10_ transformation of the *p*-value. Dotted lines on the x-axis represent −1 and 1, respectively. **(B)** Proportion and chromosomal location of identified sex-biased genes. **(C)** The expression level of sex-biased genes such as *ENSCJPG00005012300* and **(D)** *ENSCJPG00005008895*. **(E)** The x-axis represents the location of each gene on the Z chromosome and the Y-axis shows the logFC in brain tissue. The red dotted and blue dotted lines represent the average logFC of all genes and sex-biased genes only, respectively. **(F)** The expression level of *ENSCJPG00005003198*. **(G)** Homology search results demonstrate that *ENSCJPG00005003198* of quail and *ENSGALG00000051419* of chicken are orthologous genes. **(H)** Results of functional enrichment analysis of sex-biased genes in brain tissue. Black dotted line represents -log_10_(0.01). **(I)** Heatmap for representing expression level of genes related with WD repeat function.

Using a stable reference genome-based method, we identified 69 sex-biased genes, including *ZP1* (P_adj_: 3.32e-06), *NPVF* (P_adj_: 8.24e-03), and *SAG* (P_adj_: 5.36e-05), by comparing male and female samples derived from quail brain tissue (Figure 2A and Supplementary Table S3). Of the 69 genes discovered, 61 genes, including *SLC38A9* (P_adj_: 9.17e-03), *CCNH* (P_adj_: 7.28e-05), and *RPS6* (P_adj_: 3.10e-05), were upregulated in male brains, whereas only eight genes, including *KCNJ13* (P_adj_: 1.05e-02) and *TTR* (P_adj_:7.81e-05), were significantly upregulated in female brains (Figure 2B). Additionally, among the sex-biased genes discovered, 57 (82.609%) were Z-chromosomal genes, and only 12 genes were located on autosomes. Of the identified sex-biased genes, all except eight showed a female-biased pattern. In particular, two of the eight female-biased genes, *ENSCJPG00005012300* (Figure 2C) and *ENSCJPG00005008895* (Figure 2D), were lncRNAs whose functions in relation to sex differences are unknown.

Most of the sex-biased genes observed were Z-chromosome genes; simultaneously, they showed a highly male-biased pattern (Figure 2E). The average log_2_ fold change (logFC) of the 982 annotated Z-chromosome genes was 0.512. In addition, among the sex-biased genes identified, the average logFC of 57 Z chromosome genes was 0.839. This phenomenon, in which the expression levels of Z-chromosomal genes are biased toward males, is well-known evidence that avians do not have a dosage compensation mechanism. Notably, since males are the homogametic sex (ZZ), the Z chromosomal gene should be upregulated compared to females (ZW); however, the *ENSCJPG00005003198* lncRNA (P_adj_: 3.32e-06) showed a significant female-biased expression pattern (Figure 2F). As this gene is also a non-coding RNA with an unknown function, a sequence-based homolog search was performed to characterize the lncRNA based on the chicken genome. It was confirmed that the sequence was similar to that *ENSGALG00000051419* (E-value: 0.0), located in the MHM of the chicken genome (Figure 2G). Furthermore, the expression pattern of *ENSGALG00000051419* was analogous to that of *ENSCJPG00005003198* in quail (Supplementary Figure S2A).

We performed functional enrichment analysis on 69 sex-biased genes that showed sex differences in the brain tissue, resulting in seven significant functional terms (Figure 2H and Supplementary Table S4). Among these, five functional terms were related to the WD40 repeat domain (WDR), and eight genes, including *WDR41* (P_adj_: 1.58e-02), *WDR70* (P_adj_: 2.44e-03), and *WDR36* (P_adj_: 1.37e-03), were associated with this function (Figure 2I). All these genes were upregulated in the male brain, suggesting that they may be associated with male-specific phenotypes. We confirmed that these WDR function-related genes exhibited similar sex-biased patterns in chicken brains (Supplementary Figure S2B).

### Characterization of sex-biased genes in gonadal tissues

**Figure 3:**
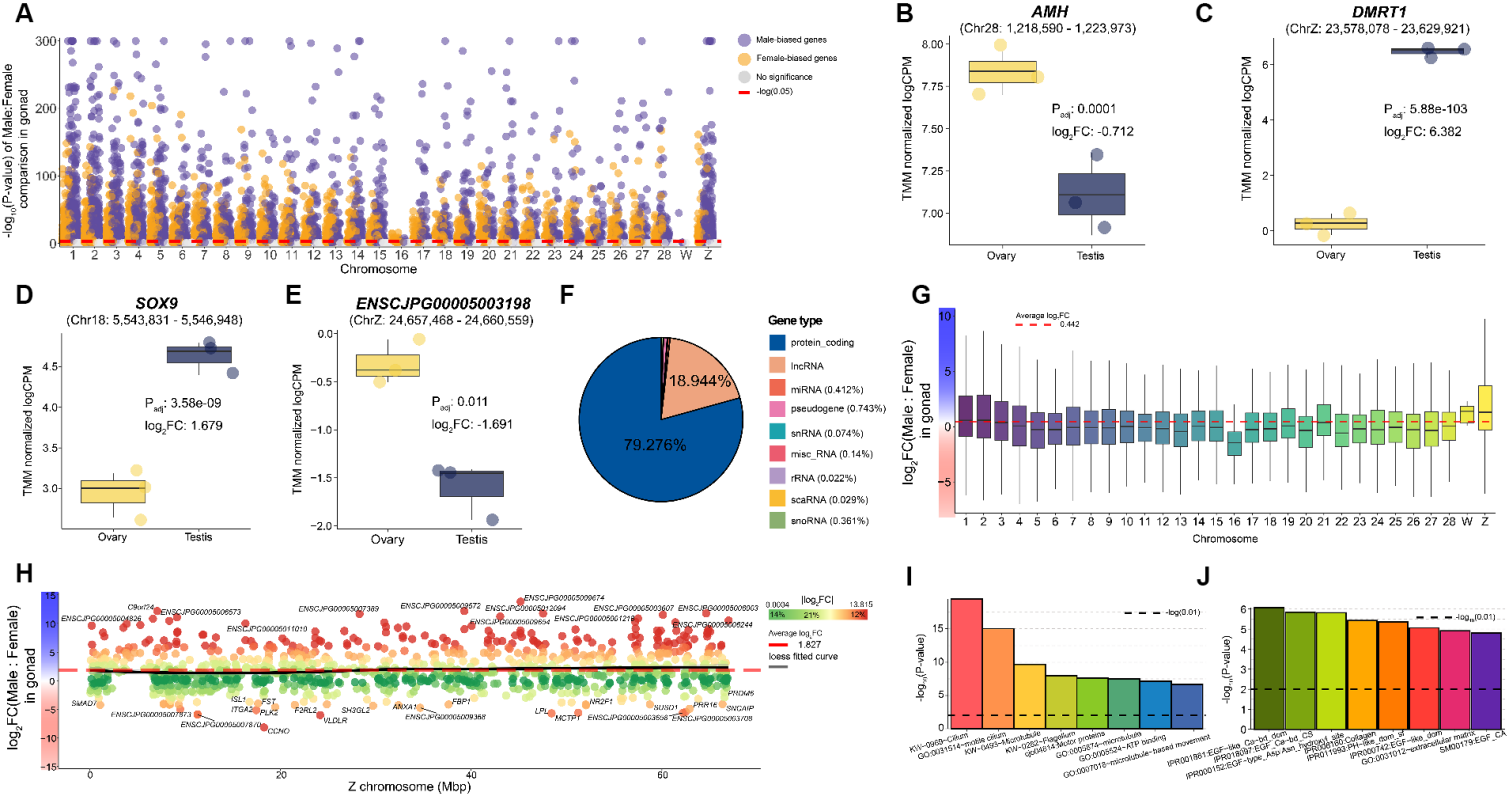
Sex-biased genes identified in quail gonad. **(A)** Sex-biased genes significantly identified in quail gonadal tissue. The x-axis represents chromosomes, and the Y-axis shows the -log_10_ transformation of the p-value. The red dotted line on the y-axis represents -log_10_(0.05). **(B)** The expression level of representative sex-biased genes such as *AMH* **(C)** *DMRT1* **(D)** *SOX9* **(E)** and *ENSCJPG00005003198.* **(F)** Proportion of sex-biased genes in gonad tissue by transcript type. **(G)** Average logFC between male and female per chromosome for all 19,224 genes in the gonad. A positive value on the y-axis indicates a male bias. **(H)** Sex-biasness of total Z-chromosomal genes in gonadal tissues. The x-axis represents the location of each Z-chromosomal gene, and the y-axis represents the logFC of each gene. The black line represents the loess fitted curve and the red dashed line represents the average logFC of Z-chromosomal genes. **(I)** Results of functional enrichment analysis of male-biased genes identified in the gonad. The black dashed line represents -log_10_(0.01). **(J)** Results of functional enrichment analysis of female-biased genes identified in the gonad.

We identified 13,593 sex-biased genes that showed differences in expression levels in gonadal tissues between the sexes (Supplementary Table S5). We confirmed that numerous sex-biased genes were distributed across the entire chromosome, compared to that found in the brain (Figure 3A). In the brain, very few sex-biased genes were autosomal; therefore, most of the discovered genes exhibited male-biased patterns, whereas the number of sex-biased genes discovered in the reproductive organs was 7,158 (52.659%) in males and 6,435 (47.341%) in females. Among the 13,593 genes, *DMRT1* (P_adj_: 5.88e-103), *SOX9* (P_adj_: 3.58e-09), and *AMH* (P_adj_: 0.0001), which are well-known sex determination-related biomarkers in chickens (Supplementary Figures S3A–C), exhibited the same expression patterns in quails (Figures 3B–D). We also found that *ENSCJPG00005003198* (P_adj_: 0.011), which was identified as a female-biased gene in the brain and a putative MHM marker in chickens (18), showed distinct expression differences in the reproductive tissues of quails (Figure 3E) and chickens (Supplementary Figure S3D). While the majority were protein-coding genes (79.276%) among the sex-biased genes identified, 2,574 lncRNAs (18.944%) showed significant differences in expression levels between the sexes in gonadal tissues (Figure 3F). The involvement of various lncRNAs in sex differences is consistent with the results of recent avian research. These results suggest that most avian species with evolutionarily similar ZW sex determination systems likely possess similar transcriptomic sexual dimorphism mechanisms.

Most studies investigating birds using ZW sex determination systems have demonstrated the absence of a global dosage compensation mechanism. Only 6,565 (36.177%) of the 18,147 autosomal genes were significantly male biased, whereas 590 of the 1,072 Z-chromosomal genes (55.037%) were male biased. The average logFC of all genes across the whole genome was 0.442, indicating that genes were expressed more in males, on average (Figure 3G). The average logFC of all Z-chromosomal genes was 1.827, indicating a strong male-biased pattern (Figure 3H). These results provide evidence supporting the fact that dosage compensation does not occur in sex chromosomal genes in the reproductive system of quail, as in many birds, including chickens.

We characterized the functions of male-biased genes identified in reproductive tissues, and 24 significant functions were revealed, including cilium (P_adj_:1.24e-18), microtubules (P_adj_:1.53e-09), and ATP binding (P_adj_: 6.93e-05) (Figure 3I and Supplementary Table S6). A total of 52 male-biased genes, including *TEKT2* (P_adj_: 3.56e-309), *RPGRIP1L* (P_adj_: 9.08e-307), and *DNAAF1* (P_adj_: 1.54e-290), were involved in cilium, a representative term highly related to male reproduction. As a result of performing enrichment analysis on female-biased genes, a total of eight significant functional terms were discovered including epidermal growth factor (EGF) (P_adj_: 0.003), collagen (P_adj_: 0.004), and extracellular matrix (P_adj_: 0.002) (Figure 3J and Supplementary Table S6). These results showed that the core functions discovered were identical to those of chicken sexually dimorphic genes.

### Tissue-specific expression patterns of sex-biased genes in Japanese quail

**Figure 4:**
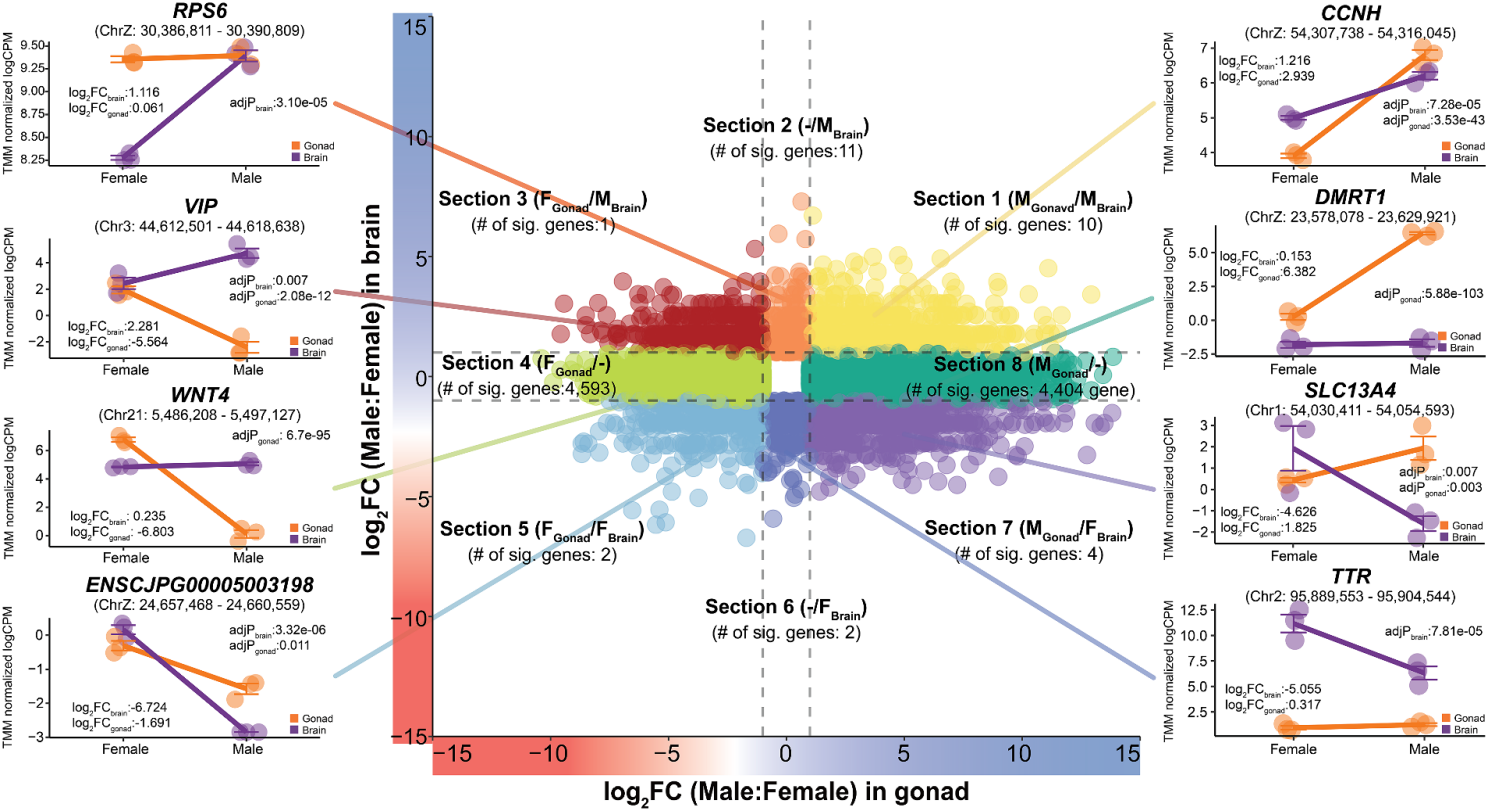
Tissue-specific expression patterns of sex-biased genes in Japanese quail. It was classified into a total of eight sections (Section 1–Section 8) to show that the expression patterns of sex-biased genes changes depending on different tissues. The x-axis represents logFC from gonadal tissue and the y-axis represents logFC from brain tissue. The entire quail gene was visualized simultaneously and the results, with additional consideration of statistical significance, are labelled in each section. The dashed lines on each axis represent −1 and 1 values.

We further analyzed the tissue-specificity of sex-biased genes found in the brain and reproductive organs, which are two representative organs with prominent sex differences. We classified sex-biased genes into eight subtypes with different directionalities based on the three types of sex bias (female- or male-biased, or no bias) observed in the two tissues (brain and gonad) with each gene’s logFC (Figure 4 and Supplementary Table S7). Among the eight subtypes, section 1 (M_Gonad_/M_Brain_) contained 716 genes showing a male-biased pattern in both tissues, of which 10 genes, including *CCNH*, *APTX*, and *NDUFS4* showed statistical significance. These male-biased genes were functionally enriched in 42 functional terms related to male reproductive ability, including sensory transduction (P: 1.82e-05), keratin (P: 1.93e-05), and the G-protein coupled receptor signaling pathway (P_:_ 2.33e-05) (Supplementary Table S8). Conversely, there were 329 genes for which a female-biased pattern was observed in both tissues, of which only two genes, *ZP1* and *ENSCJPG00005003198*, were statistically significant (F_Gonad_/F_Brain_; Section 5).

A total of 211 genes classified in Section 2 (−/M_Brain_) showed a male-biased pattern only in the brain. Among them, 11 statistically significant genes were discovered, including *RPS6*, *SLC38A9,* and *RPS23*. These genes were functionally enriched in six significant terms, including neuroactive ligand-receptor interactions (P: 0.0006) and G-protein-related pathways. In contrast, only two genes, *SAG* and *TTR*, were significant among the 227 genes belonging to section 6 (−/F_Brain_), which showed a female-biased pattern only in the brain. These genes play important roles in olfactory-related functions and thyroid hormone regulation, which are characteristics of the female quail brain.

Section 8 (M_Gonad_/−) and section 4 (F_Gonad_/−), subgroups of genes showing a male- or female-biased pattern only in the reproductive organs, contained 4,775 and 4,883 genes, respectively. Most of these, 4,404 male-biased genes including *DMRT1* and *SOX9,* and 4,593 female-biased genes, including *WNT4* and *FOXL3*, were also statistically significant. The 4,404 genes showing a male-biased pattern in the gonads alone were functionally enriched in 22 significant functions related to male fertility, such as motile cilium (P: 6.99e-17), motor proteins (P: 1.66e-08), and microtubules (P: 3.58e-08). In contrast, 4,593 female-biased genes of significance only in the gonads were functionally enriched for 157 different functions, including helical (P: 4.92e-13), EGF (P: 3.13e-07), and extracellular matrix (P: 5.31e-06). These results indicate that a significant number of transcripts are involved in reproductive functions in different sexes and that this is highly tissue-specific.

We observed genes belonging to two sub-groups, section 3 (F_Gonad_/M_Brain_) and section 7 (M_Gonad_/F_Brain_), which showed different directional sex-biased patterns in the two tissues. The only significant gene showing a female-biased pattern in reproductive tissues, but a male-biased pattern in the brain, was *VIP*. In section 7, which is the opposite of section 3, four genes, *KCNJ13, SLC13A4*, *ENSCJPG00005008895,* and *ENSCJPG00005012300* were found to be statistically significant. Genes whose sex-specific differential expression patterns are completely reversed depending on the tissue, which represent sex differences, and at the same time, show very strong tissue specificity. This tissue specificity of sex-biased genes in quails was also observed in chickens (Supplementary Figure S4).

### Expression profiling of genes related sex determination during embryonic development

The expression patterns of the sex-biased genes discovered in this study were similar to those in chickens, therefore, we further hypothesized that they would also show similar expression patterns during the embryonic period. To explore this hypothesis, we performed a cross-species integrated analysis based on embryonic RNA-seq data collected from quails and chickens. We first identified 12,786 orthologous genes (Supplementary Table S9) for integrated cross-species analysis, and then investigated the differences in the expression patterns of all genes at the Eyal-Giladi and Kochav (EGK) embryonic developmental stages.

**Figure 5:**
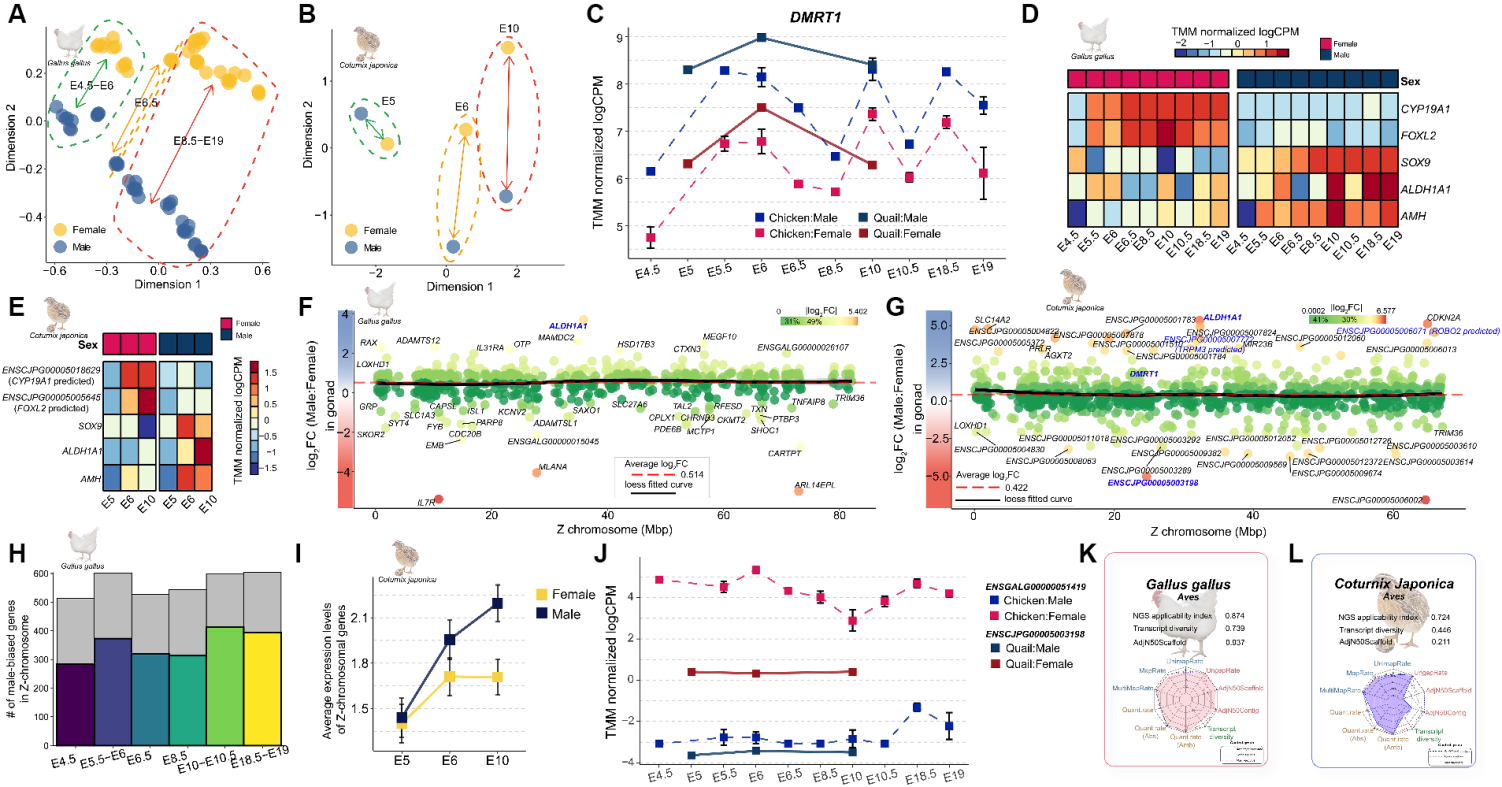
Expression profiling of genes involved in sex determination during embryonic development in Japanese quail. **(A)** The MDS plot indicates that the overall sex-differences in gene expression increase as chicken embryonic development progresses. **(B)** Quail similarly shows that overall sex differences in gene expression increase as embryonic development progresses. **(C)** Changes in the expression pattern of *DMRT1* during embryonic development stages E4.5–E19 in quail and chicken. **(D)** Expression patterns of known major sex-determining genes during embryonic development in the chicken. **(E)** in the quail. **(F)** Overall sex-biased pattern of Z-chromosomal genes in chicken. The red dashed line represents the average logFC of Z-chromosomal genes and the black line represents the Loess fitted curve. **(G)** Overall sex-biased pattern of Z-chromosomal genes in quail. Genes shown in bold blue are orthologous to sex-determining genes on the Z chromosome known from previous avian research. **(H)** Proportion of male-biased genes identified in during embryonic developmental stages of chicken. **(I)** Average expression patterns of Z-chromosomal genes in quail embryos. **(J)** Expression pattern of the *ENSCJPG00005003198* in quail and its orthologue, *ENSGALG00000051419* in chicken. **(K)** NGS applicability index for quality of reference genome and gene annotation in chicken. The fuller the polygon, the better the quality. **(L)** NGS applicability index in quail.

There was a relatively small difference in expression levels between male and female at E4.5 developmental stage when sexual differentiation began in both quail (Figure 5A) and chickens (Figure 5B), compared to those in later stages of embryonic development. In chickens, as the embryonic developmental stage progressed, the difference between males and females in the expression patterns of whole transcripts gradually increased (Supplementary Table S10). We further examined the expression patterns of *DMRT1* (Figure 5C), the master gene for sex determination in avians, and found that it was consistent with the results of previous studies on chickens. In a recent study, the expression level of *DMRT1*, which was confirmed to have sex-determining potential using CRISPR technology (23), was male-biased throughout the embryonic stages in both species. Sexual dimorphic genes that play an important role in sex determination, such as *SOX9*, *AMH*, and *ALDH1A1*, showed a significantly male-biased pattern in chicken embryos (Figure 5D), and the same male-biased pattern was also observed in quails (Figure 5E). While performing a cross-species integrated analysis of quail and chicken embryos, we discovered that *FOXL2* and *CYP19A1*, which are known to play important roles in sex determination, were missing annotations in the quail reference genome. As a result of searching for the corresponding genes in the *Coturnix japonica 2.0* reference genome through a BLAST homolog search, it was confirmed that *FOXL2* and *CYP19A1* genes were annotated as *ENSCJPG00005005645* (E-value: 0.0), an lncRNA, and *ENSCJPG00005018629* (E-value: 0.0), a protein-coding gene (Supplementary Figure S5, S6). Although misannotated as an lncRNA and a protein-coding gene, respectively, they showed significantly high sequence similarity to *FOXL2* and *CYP19A1* genes in chickens, and showed the same strong female-biased pattern in both species (Figures 5D,E). These results suggest that the sex-determination mechanisms of quail and chicken, which are evolutionarily similar, will be very similar. The fact that sex-linked genes for sex determination were misannotated suggests that the quality of the reference genome and gene annotation in Japanese quail is still incomplete compared to that of the model species.

Next, we investigated the expression patterns of Z-chromosomal genes, which are key factors in sex determination during embryonic development. There is no general dosage compensation in which some regions are inactivated to match the expression levels of Z-chromosomal genes derived from the ZZ (male) chromosome with those of ZW (female) individuals in birds with a ZW sex-chromosomal system. We reexamined whether this claim was true for quail embryos at the sexual differentiation stage. The average logFC of all Z-chromosomal genes across all embryo developmental stages was 0.514 (Figure 5F) for chickens and 0.422 for quails (Figure 5G), which were commonly male-biased on average. In addition, because the Loess fitted curve converged to the average line in both species, it was reconfirmed that there was no global dosage compensation mechanism in the Z chromosome. Examination of the number of Z-chromosomal male-biased genes in the two species revealed that an average of 61.586% of the genes were male-biased throughout embryonic development (Figure 5H). In contrast, there was no difference between quail males and females at the E5 stage, which is the start of sexual differentiation, but at the E10 stage of development, 76.763% of genes showed a male-biased pattern, similar to chicken embryos (Figure 5I). We examined the expression pattern of *ENSCJPG00005003198* lncRNA, an MHM regional gene that plays an important role in sexual dimorphism (18), and found that the same female-biased pattern was observed not only in the brain and gonad tissues of adult quails but also throughout the entire embryonic development stage (Figure 5J). All the indicators examined from various perspectives support the absence of a dosage compensation mechanism for Z-chromosomal genes in quail, similar to that observed in chickens.

While most results from the cross-species analysis of chicken and quail support the hypothesis that the mechanism of sexual dimorphism in quail is likely to be similar to that in chickens, we found that a significant number of lncRNAs in the Z-chromosomal genes of quail showed relatively strong sex bias compared to the expression pattern in chickens (Figures 5F,G). This can potentially be interpreted in two different directions: (1) there is indeed a quail-specific mechanism by which Z-chromosomal lncRNAs are involved in sexual dimorphism; and (2) this is a technical limitation that arises because the reference genome and gene model of quail, are incomplete. To determine which of the two interpretations was valid, we evaluated the reference genomes and gene annotations of chicken and quail based on a recently developed NGS applicability index (25). The NGS applicability index for the reference genome and gene annotation of chicken was 0.874 (Figure 5K), whereas that for quail was 0.724 (Figure 5L). In particular, transcript diversity, which is a quality indicator for gene annotation among the components that constitute the NGS applicability index, was 0.739 and 0.446 in chickens and quail, respectively, indicating that gene annotation in quail is still incomplete. Moreover, considering that genes that are key to sex determination, such as *FOXL2* and *CYP19A1*, which were previously discovered, have still been misannotated in the quail genome (Figures 5D,E), it is more reasonable to interpret the discovery as a result of technological limitations rather than the interpretation that quail-specific sexual dimorphic mechanisms involve Z-chromosomal lncRNAs.

## Discussion

The identification of genes associated with sexual dimorphism in quail has so far been based on a *de novo* assembly approach owing to the late release of the official reference genome in 2016. Most bioinformaticians empirically believe that reference genome-based methods are more accurate than reference-free methods; however, to our knowledge, this has never been verified in quail. In this study, we demonstrated this academic question that the reference guide method was superior in all aspects, at least in quail (Supplementary Figure S1). By 2024, only the reference genomes of 317 biological species will be officially released from the Ensembl database (27); however, these are only a few of the approximately 850 million species currently estimated to exist (46). Therefore, for the vast majority of biological species, reference genomes and gene annotations have not yet been established, and *de novo*-based approaches must be used. Although our results showed that the reference-based method was significantly more stable than the reference-free method only in quail, it is presumed that the same outcomes may be obtained in other species. We found evidence in a comparative analysis of reference-free and reference-based methods in *Rhodnius prolixus* (47). Additionally, lower expression levels of specific transcripts and longer or more complex forms of transcripts in the genome exacerbated the limitations of the reference-free method (48). However, no study has systematically compared different experimental factors, such as the structure or sequencing of these transcripts, with different assembly methods in different species to generalize whether reference genome-based methods are more reliable. We believe that this will be more clearly demonstrated in the near future through systematic benchmark studies comparing reference-guided and reference-free methods across multiple species under different experimental conditions.

In this study, we identified several sex-biased genes that represent masculinity in quail. The *RPS6* male-biased gene in quail brain tissue (Figure 2A) was not affected by dosage compensation in embryonic fibroblast cells from seven avian species (17). In addition, *RPS6* showed a strong male-biased pattern in the embryonic developmental period of chickens and quails (Supplementary Figure S7) and has a substantial impact on brain development during the embryonic period of the zebra finch (49). In chicken embryonic fibroblast cells, this *RPS6* gene plays a role in cell cycle regulation including cell division and proliferation, indicating its importance in the maintenance and progression of the cell cycle (50). Also, in the zebra finch, this gene showed male- biased pattern in song region of the brain, which implicated that this gene was related with the vocal learning in zebra finch (49). Therefore, we confirmed that the *RPS6* is a sex-linked gene that shows a common male-biased pattern in all avian species within the ZW sex system, including quail. *CCNH* was a common male-biased gene in the brain and reproductive organs of quails (Figure 4) and as well as chickens, even during the embryonic period (Supplementary Figure S8). This gene plays an important role in brain development in early chicken embryos (51, 52). This suggests that *CCNH* plays an important role in functions commonly associated with male brain characteristics in birds, including chickens and quail. *DMRT1* has a role in gonadal sex differentiation in male chickens (21), which has already been verified at the individual level using the CRISPR-Cas9 (23). We confirmed that *DMRT1* also showed a strong male-biased pattern in quail at the embryonic stage of sex determination (Figure 5C) and in the testis (Figure 4). Similarly, the *SOX9* gene, which is known to play a key role in chicken and duck testis development (53, 54), was also commonly male-biased during chicken and quail embryonic development (Figures 5D,E) and was confirmed to be upregulated in the testes of adult quails (Figure 3D).

We also identified genes commonly associated with quail femininity, from embryonic stages to adult tissues. The female-biased pattern of the *ZP1* gene gradually becomes stronger from the embryonic stage, when sex was determined in both quail and chicken (Supplementary Figure S9). After differentiation into brain and gonad tissues, *ZP1* maintained a strong female-biased pattern, regardless of the tissue. *ZP1* is highly expressed in the liver tissues of laying females (55) and has a highly correlation with estrogen levels (56). In addition, it is widely known that there is a high association with the diversity of the egg envelope in avian species, including quail and chicken (57, 58), which is key to supplying proteins to humans during breeding. The strong female-biased pattern of *ZP1* was confirmed in quail as a key gene with conserved functions in females, including reproductive activity in birds. Simultaneously, this study also identified that the female bias of *ZP1* gradually strengthened after sex determination. Notably, we found an lncRNA, *ENSCJPG00005003198*, which showed a female-biased pattern from the embryonic period (Figure 5J) to adult tissue (Figures 2F, 3E). We also found that this quail lncRNA had high sequence identity with chicken *ENSGALG00000051419* (Figure 2G). *ENSGALG00000051419* lncRNA was identified as one of the genes responsible for incomplete feminization in transgenic experiments through *DMRT1* gene disruption in chickens, and *in situ* hybridization verified that the female-biased pattern was maintained independent of *DMRT1* expression (23). *ENSGALG00000051419* is located in the MHM region of the Z chromosome and differences in expression levels occur through significant differential methylation between the two sexes (59). Therefore, female sex-related functions are not determined by the spontaneous expression of *ENSGALG00000051419*; the non-translated RNA sequences transcribed in the MHM region are female-biased due to sex-specific methylation in the genetic region. We confirmed the removal of the *ENSGALG00000051419* marker from a recently updated chicken *GRCg7b* model, which is one of the key pieces of evidence supporting our interpretation (Supplementary Figure S10). We expect that this observation will be clarified when accurate transgenic experiments on methylation levels become possible in the future. We confirmed once again that the expression patterns of transcripts showing male or female bias in both embryonic and adult tissues were identical in the evolutionarily close quail and chicken.

We investigated the dosage compensation mechanism of the Z chromosome, an important axis of sexual dimorphism in quail species. There was no global dosage compensation mechanism in either the brain tissue (Figure 2E) or reproductive organs (Figure 3H) of adult quails. These results are consistent with those of studies on chickens that have investigated the presence or absence of dosage compensation in birds (60, 61). We also confirmed that there was no overall dosage compensation in either quail or chicken during embryonic development (Figures 5F,G), which is consistent with previous avian studies on dosage compensation (11, 16). This incomplete dosage compensation has been consistently reported in other species with ZW sex-determination systems. For example, *Corvus corone* (62), a bird with a ZW system, *Heloderma suspectum* (63), and *Bombyx mori* (64) have incomplete dosage compensation. In contrast, *Manduca sexta*, a species with a ZW sex determination system, showed complete dosage compensation (65), while *Apalone spinifera* showed a complete dosage compensation pattern dependent on temperature (66). A review of research on dosage compensation in different species showed that the completeness of dosage compensation is not determined by the ZW sex determination system. However, this study suggests that evolutionarily close avian species, such as quail, chicken, and *C.corone* have a similar incomplete dosage compensation mechanism.

We observed sex-related genes in which the sex-biased pattern of gene expression changed depending on the tissue and/or specific time points of embryonic development. For example, the *AMH* gene is male-biased in both male chickens and quails during the embryonic stages (Figure 5D,E), whereas it was female-biased in the adult reproductive organs (Figure 3B). Also, this female-biased gene expression pattern in reproductive organs was observed in chickens as well (Supplementary Figure S3A). This observation is consistent with previous findings that *AMH* shows a male-biased pattern during embryonic development in quails (67) and is upregulated in the ovaries of adult quails (34).In addition, the results of previous studies on chickens showed the same trend as our findings (68–70), supporting the claim that the expression pattern is evolutionarily conserved between the two species. *AMH* plays an important role in the differentiation and development of male reproductive organs in avian embryonic studies (67, 69, 71), and in adults, it is associated with female reproductive functions, including follicular development (72, 73). Therefore, this study showed that *AMH* has different sex-biased patterns throughout the embryonic and adult periods in both quail and chickens. Even for *WNT4*, which is widely known as a sex-linked gene, we found that the expression pattern was reversed depending on the time point in quails and chickens. The expression level of *WNT4* showed a male-biased pattern until stages E6 and E6.5 at the time of sex determination in quail and chickens, respectively, but reversed to a female-biased pattern after sex determination (Supplementary Figure S11 and Figure 4). This finding is consistent with the results of previous independent chicken embryo development studies (74–76) and provides further evidence that there is an evolutionarily conserved sex determination mechanism in quail and chicken.

As sex determination is a common attribute of all living organisms, it has been demonstrated that genes identified as directly associated with this process exhibit comparable gene expression patterns in evolutionarily analogous quails and chickens. On the other hand, most other genes showed heterogeneous expression patterns between the two species. As an example, the *VIP* gene showed a male-biased pattern in the quail brain (Figure 4 and Supplementary Figure S12A) but was a female-biased gene in the chicken brain (Supplementary Figure S12B). On the other hand, both adult quails and chickens showed the same expression pattern of female-biased genes in the gonads. The male-biased pattern of the *VIP* gene in the quail brain has been technically verified by qRT-PCR in an existing study (29). In addition, the *VIP* showed a female-biased pattern in the chicken brain, which is also consistent with existing transcriptome research (76). The *VIP* is known to be involved in energy balance and appetite regulation in brain tissue (77) and is also known to affect the balance and behavior of the autonomic nervous system in birds (78, 79). Therefore, we speculate that the sex-biased pattern of *VIP* may vary between the two species, as it involves a wide range of functional regulation, including different behaviors in the different species. We further investigated whether heterogeneous sex-biased gene expression patterns in *VIP* also occur during embryonic development (Supplementary Figure S12C). In quail, *VIP* is female-biased during embryonic development, whereas in chickens it shows a male-biased pattern until the development of the reproductive organs is complete (from E4.5 to E10.5). This observation was consistent with the findings of previous studies that the *VIP* is male-biased during the early embryonic process in chickens (74, 77). We speculate that the opposing expression patterns found between these two species may be due to the different embryonic developmental stages in the two species. The average embryonic development period for chickens is known to be 21 days, while the embryonic period for quails is approximately 16 days (78). In addition, the development of the quail’s reproductive organs begins on day 3, earlier than in chickens (79, 80). Thus, the female-biased pattern observed at quail embryonic days E6 and E10 in this study is presumed to be the same as that at chicken embryonic days E12 to E19 (Supplementary Figure S12C). If a noninvasive method to obtain early quail embryos is developed, we expect to be able to technically verify this observation.

Taken together, these results suggest that the sex-biased pattern can be reversed depending on the embryonic developmental stage and/or tissue, as sex-biased genes perform various functions *in vivo*. Additionally, observations of these time-point and/or tissue-specific sex-biased genes have been identified not only in quail, but also in chickens, revealing that this is a common phenomenon in evolutionarily close avian species. Notably, the variable expression patterns of these sex-biased genes may be a major obstacle in breeding and transgenic experiments. For example, only a single approach for suppressing or activating the expression of a specific gene can be considered when conducting transgenic research or genetic breeding using modern biotechnology. Evidence for this claim comes from *DMRT1* disruption experiments based on CRISPR/Cas9 genome-editing technology performed on early embryos for sex determination in chickens (23, 81). In these studies, primary feminized chickens were successfully generated by suppressing *DMRT1* at the early embryonic stage, but failed to obtain secondary female traits, such as ability to reproduce. Although the causes of these transgenic outcomes of incomplete sex determination are presumed to be complex, we suggest that a critical factor is a change in the direction of the sex-biased pattern at a specific point in time and/or tissue. If a transgenic or breeding method that can control the gene expression levels of a specific tissue in real time can be developed in the future, we expect that the proposed hypothesis will be confirmed.

In this study, we collected as much publicly available RNA-seq data as possible to identify genes related to sexual dimorphism and sex determination in quail. However, there are practical limitations in that it was not possible to collect data at the chicken level in terms of data volume and diversity. To compensate for this practical difficulty, we conducted an integrated cross-species analysis based on the premise that the evolutionarily close quail and chicken would share most of the mechanisms of sexual dimorphism. We demonstrated that most of the mechanisms of sex determination and expression patterns of sex-biased genes were similar between the two species. While this study has the realistic limitation of not being able to identify a quail-specific mechanism of sexual dimorphism that differs from that of chickens, we expect that this will be verified in the future as transcriptomic data for quail accumulate over time. The findings of this study on the different sexual dimorphisms in quail will fundamentally improve our understanding of the mechanisms of sex determination in birds and will be important for applications that benefit humans, such as breeding and transgenesis, for egg production.

## Data availability statement

All analysis code and preprocessed data included in this study is available in the github repository (https://github.com/ABCLabDB/JapaneseQuail_RNA_Seq). Accessions to all collected RNA-seq raw data are listed in Supplementary Table S1.

## Ethics statement

Ethical approval was not required for the study involving animals in accordance with the local legislation and institutional requirements because this is a meta-analysis-based study conducted by collecting data previously conducted with ethical commitment approval.

## Author contributions

SiP: Conceptualization, Data curation, Formal analysis, Investigation, Methodology, Project administration, Resources, Visualization, Writing – original draft, Writing – review & editing. JK: Visualization, Writing – review & editing. JL: Visualization, Writing – review & editing. SJ: Visualization, Writing – review & editing. SuP: Funding acquisition, Supervision, Writing – review & editing. JHL: Funding acquisition, Supervision, Writing – review & editing. KY: Writing – review & editing. SW: Writing – review & editing. JH: Supervision, Writing – review & editing. MS: Conceptualization, Formal analysis, Funding acquisition, Investigation, Methodology, Project administration, Resources, Supervision, Validation, Writing – original draft, Writing – review & editing.

## Footnotes

^1^https://github.com/ncbi/sra-tools
