# Supplementary material for: RNA sequencing analysis of sexual dimorphism in Japanese quail
Source: Front Vet Sci. 2024 Jul 22;11:1441021. doi: 10.3389/fvets.2024.1441021 (PMC11299063; doi:10.3389/fvets.2024.1441021)
Supplement: Supplementary file 1 [file Data_Sheet_1.docx]

Supplementary Material

## Supplementary Figures

[Supplementary Figure S1. Reference genome-based approaches can more accurately identify sex-biased genes 2](#_Toc170709220)

[Supplementary Figure S2. Technical verification based on chicken RNA-seq data for sex-biased genes identified in quail brain 3](#_Toc170709221)

[Supplementary Figure S3. Expression levels of representative sex-biased genes identified in gonadal tissues of chicken 4](#_Toc170709222)

[Supplementary Figure S4. Tissue-specific expression patterns of sex-biased genes in chicken 5](#_Toc170709223)

[Supplementary Figure S5. The result of the homolog search for *ENSCJPG00005005645* in the GRCg7b genome 6](#_Toc170709224)

[Supplementary Figure S6. A homolog search of the *ENSCJPG00005018629* sequences in the GRCg7b genome 7](#_Toc170709225)

[Supplementary Figure S7. Expression level of *RPS6* in embryonic stage of quail and chicken 8](#_Toc170709226)

[Supplementary Figure S8. Expression level of *CCNH* in embryonic stage of chicken and quail 9](#_Toc170709227)

[Supplementary Figure S9. The expression level of *ZP1* in quail and chicken 10](#_Toc170709228)

[Supplementary Figure S10. The result of homolog search for *ENSCJPG00005003198* on GRCg7b genome 11](#_Toc170709229)

[Supplementary Figure S11. The expression level of *WNT4* in the embryonic stage of quail and chicken 12](#_Toc170709230)

[Supplementary Figure S12. The expression level of *VIP* chicken and quail 13](#_Toc170709231)


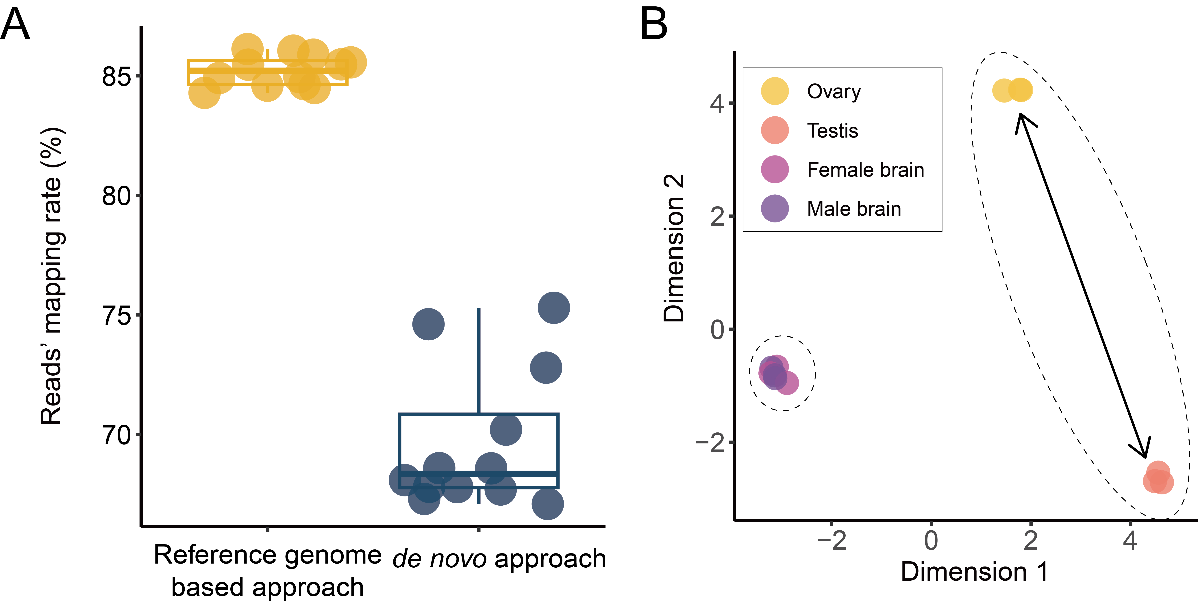


Supplementary Figure S1. Reference genome-based approaches can more accurately identify sex-biased genes

**(A)** Differences in mapping rates between reference genome-based approaches and *de novo* assembly methods. **(B)** MDS plot based on expression level quantified using a reference genome-based method. Arrows indicate gene expression differences between male and female.


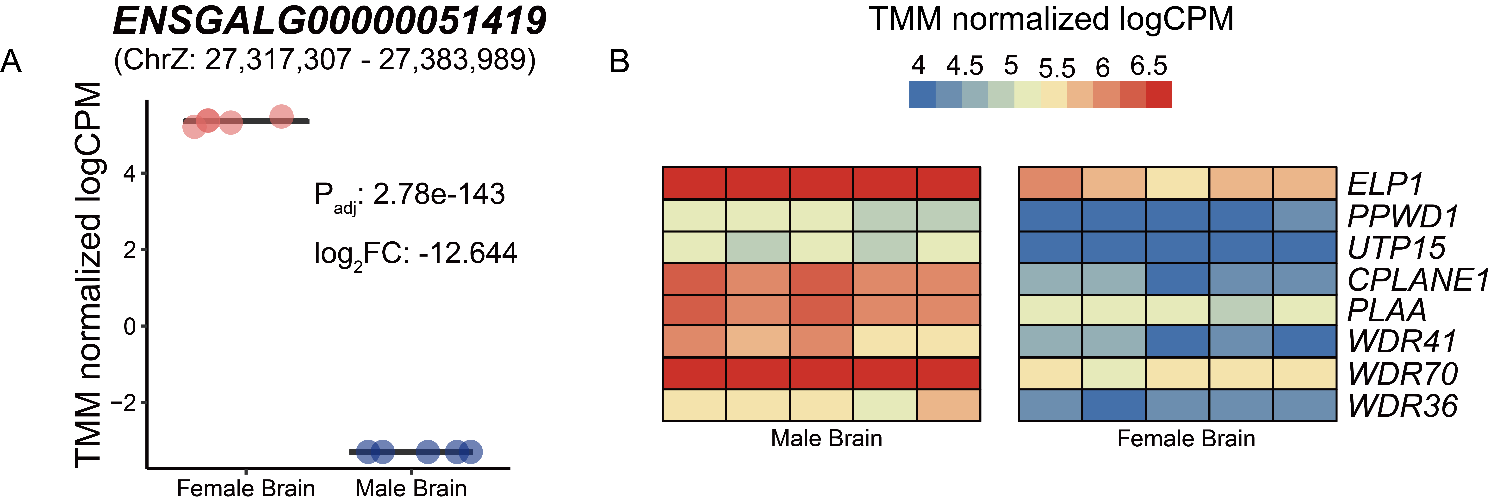


Supplementary Figure S2. Technical verification based on chicken RNA-seq data for sex-biased genes identified in quail brain

**(A)** Expression level of *ENSGALG00000051419*, an orthologous gene of *ENSCJPG00005003198*, in chicken brain tissue, both of which belong to the MHM region. **(B)** Investigation of expression patterns of sex-biased genes related WDR function identified by analyzing RNA-seq data in quail’s brain based on chicken RNA-seq data.


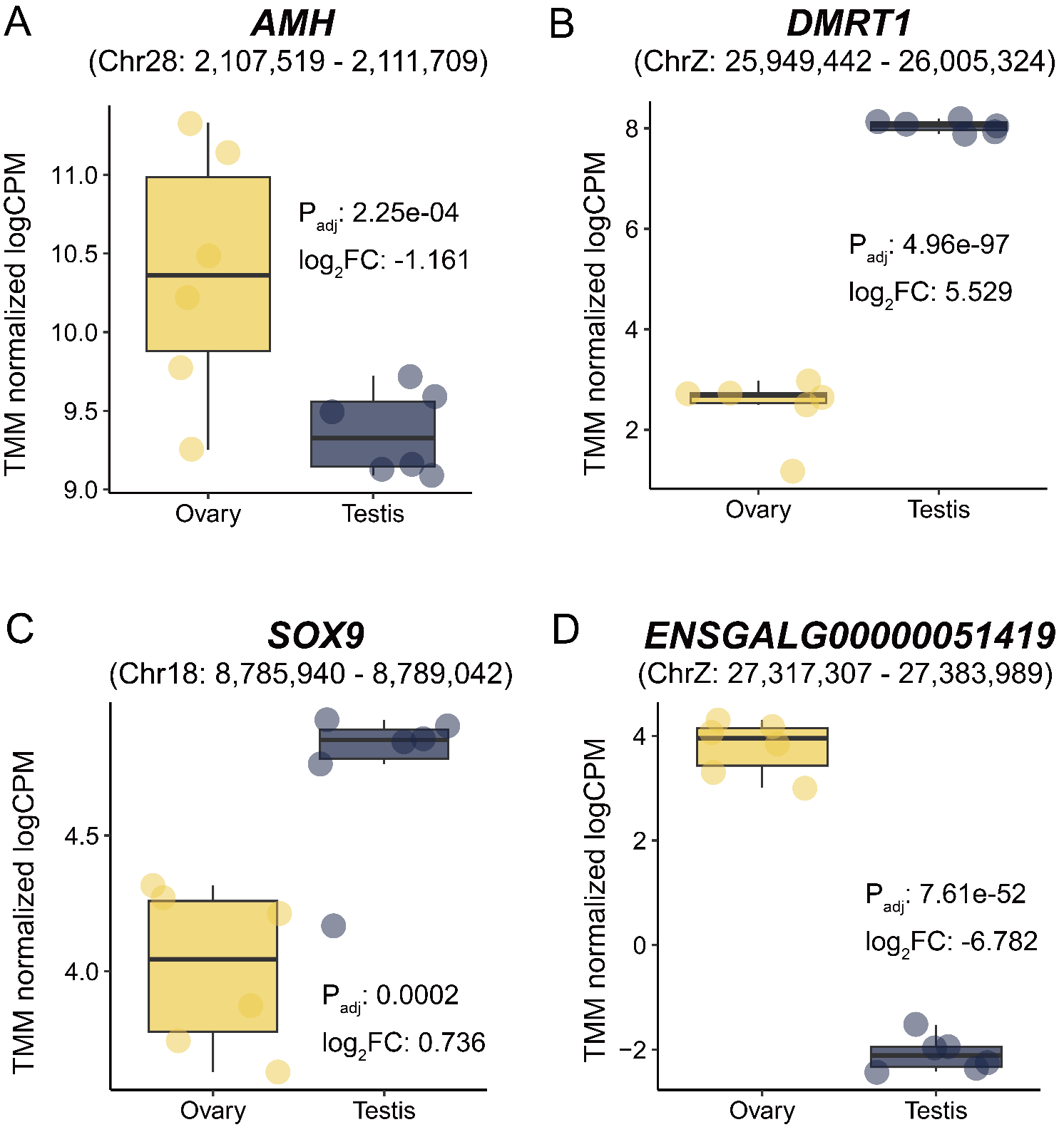


Supplementary Figure S3. Expression levels of representative sex-biased genes identified in gonadal tissues of chicken

Technical verification based on chicken RNA-seq data for representative sexual dimorphic genes identified as sex-biased genes in quail gonadal tissues. It was confirmed that both quail and chicken sex-biased orthologues showed the same expression pattern **(A)** in *AMH* gene. **(B)** in *DMRT1* gene. **(C)** in *SOX9* gene. **(D)** in *ENSGALG00000051419* lncRNA that is orthologue of *ENSCJPG00005003198* lncRNA in quail, both of which belong to the MHM region.


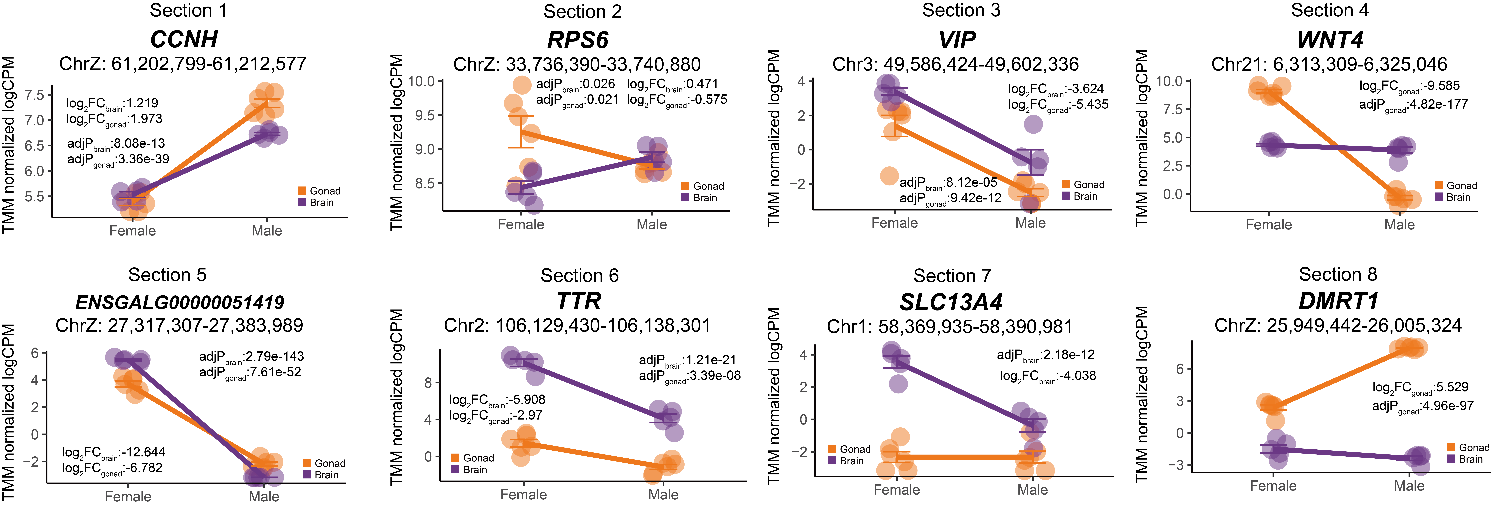


Supplementary Figure S4. Tissue-specific expression patterns of sex-biased genes in chicken

The Y-axis represents TMM-normalized logCPM. The orange line indicates gene expression in gonadal tissue, while the purple line represents the gene expression level in brain tissue.


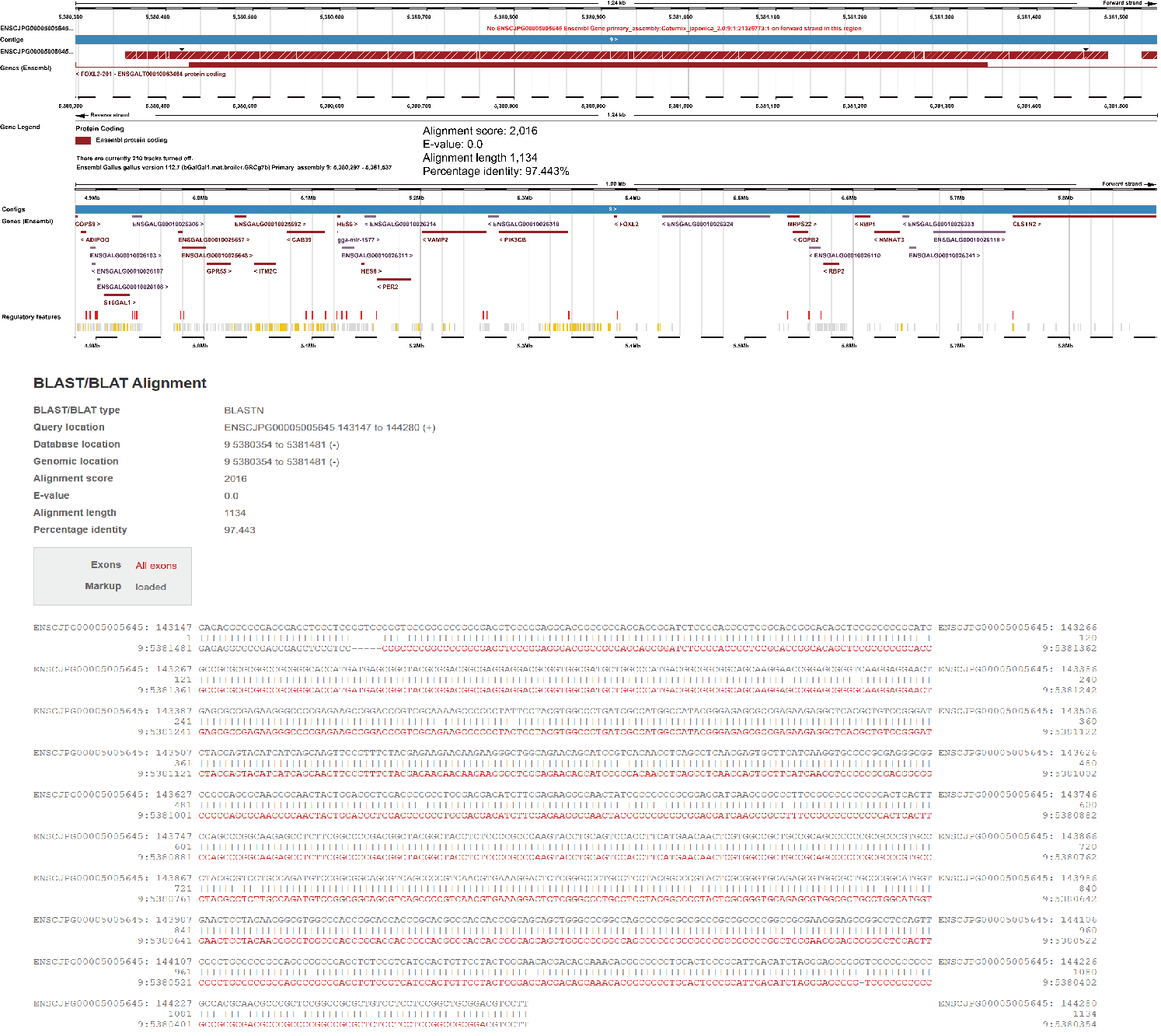


Supplementary Figure S5. The result of the homolog search for *ENSCJPG00005005645* in the GRCg7b genome

*ENSCJPG00005005645* is orthologous genes of the *FOXL2* (Chr9: 5,380,354-5,381,481) gene in the GRCg7b genome.


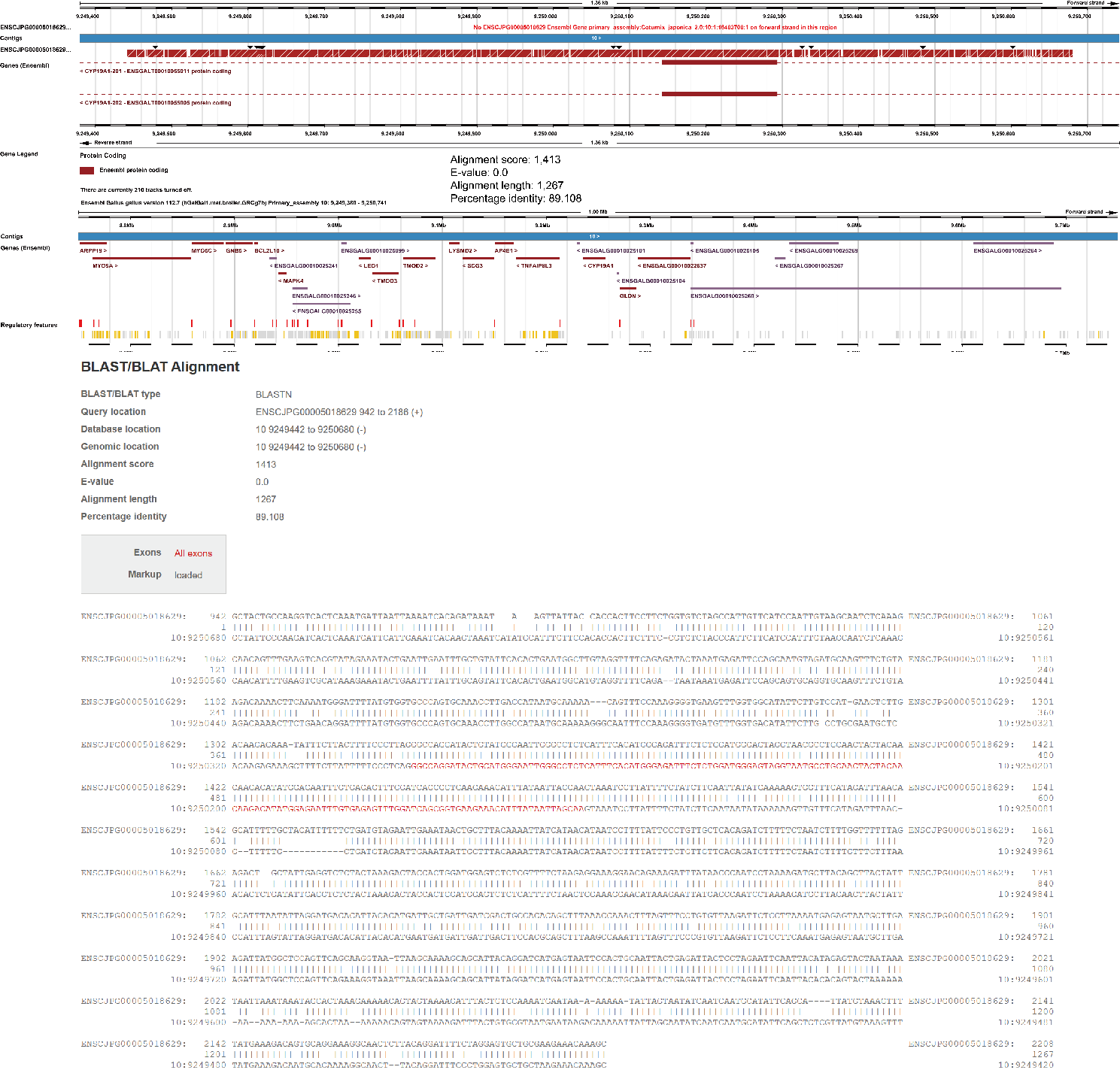


Supplementary Figure S6. A homolog search of the *ENSCJPG00005018629* sequences in the GRCg7b genome

A homology search revealed that they share similarities with the *CYP19A1* (Chr10: 9,249,442-9,250,680) gene sequences within GRCg7b.


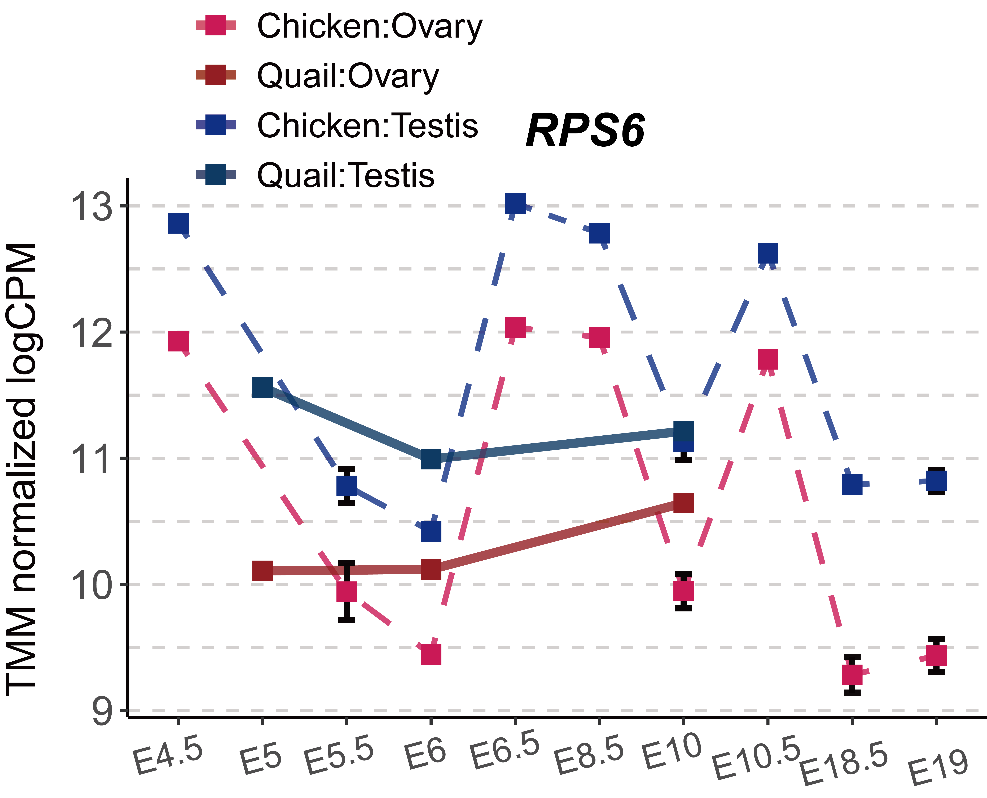


Supplementary Figure S7. Expression level of *RPS6* in embryonic stage of quail and chicken

The X-axis represents the embryonic developmental stages of birds from E4.5 to E19, as defined by Eyal-Giladi and Kochav. The Y-axis represents the expression level of the *RPS6* gene.


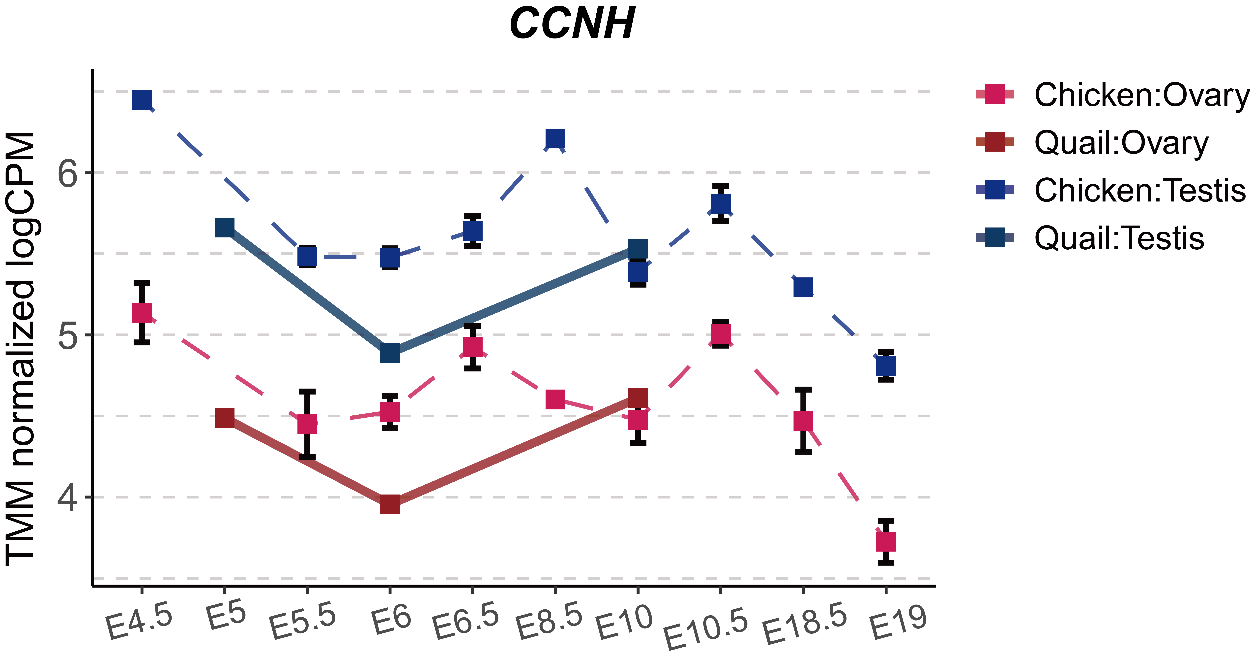


Supplementary Figure S8. Expression level of *CCNH* in embryonic stage of chicken and quail

The X-axis represents the embryonic developmental stages of birds from E4.5 to E19, as defined by Eyal-Giladi and Kochav. The Y-axis represents the expression value of the *CCNH* gene.


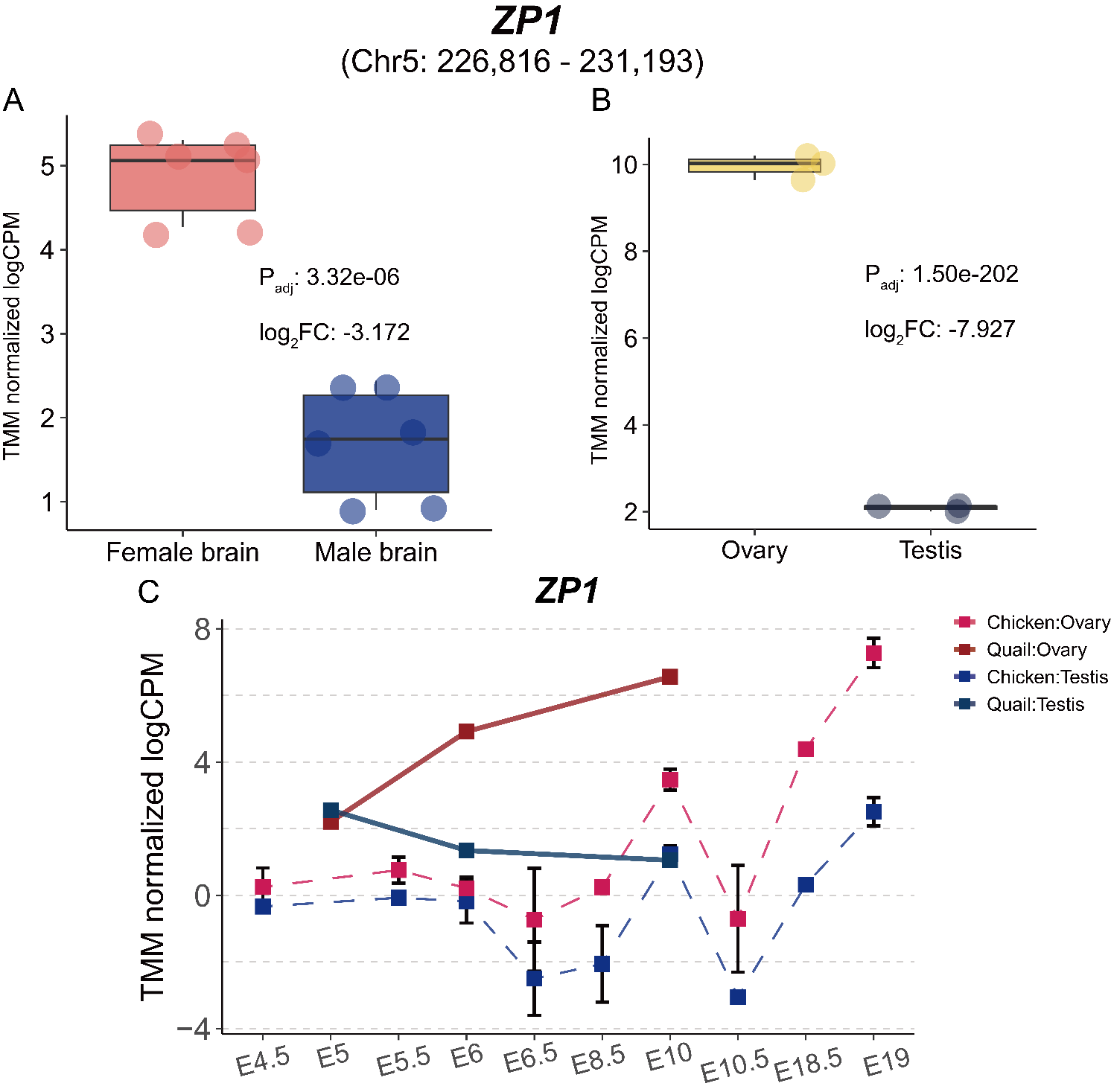


Supplementary Figure S9. The expression level of *ZP1* in quail and chicken

**(A)** The expression level of *ZP1* in brain tissue of quail. **(B)** The expression level of *ZP1* in reproductive organs of quail. **(C)** The line plot shows the gene expression level during the embryonic stages of chickens and quails. The X-axis represents the embryonic developmental stages of birds from E4.5 to E19, as defined by Eyal-Giladi and Kochav. The Y-axis represents the expression value of the *ZP1* gene.


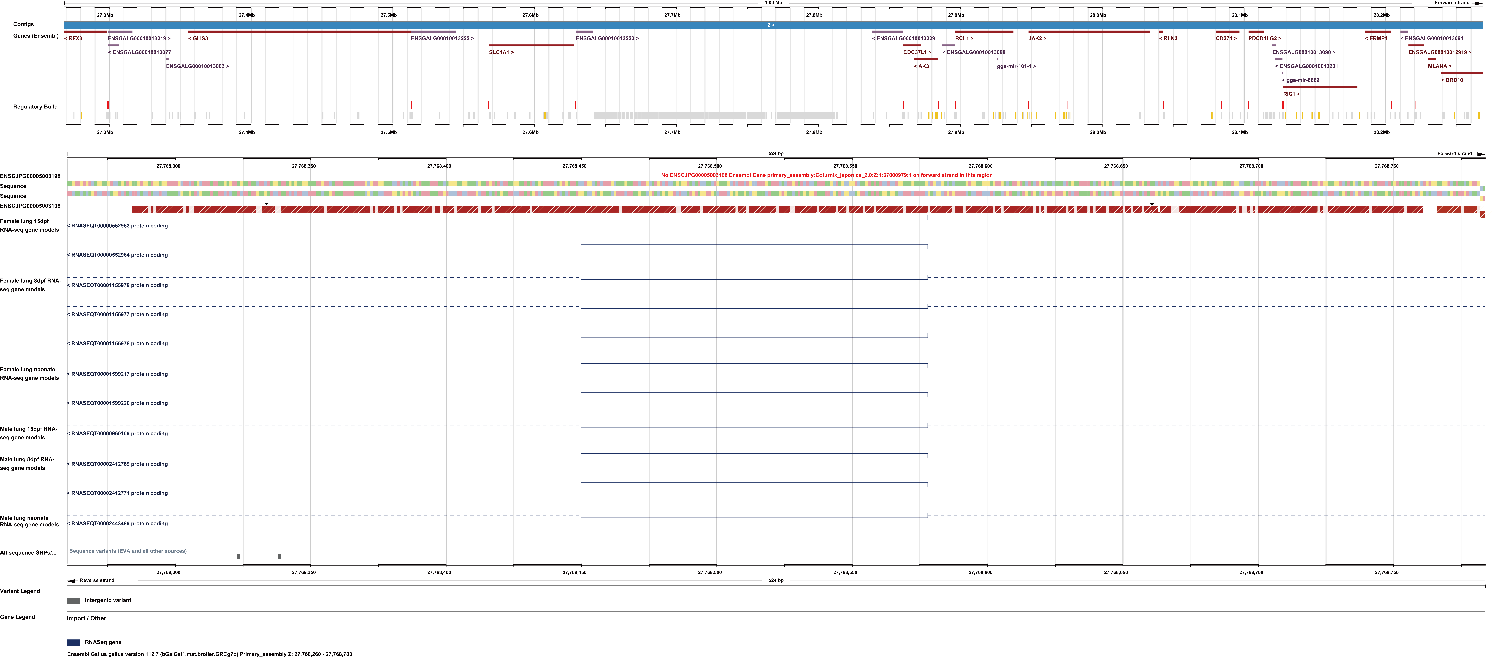


Supplementary Figure S10. The result of homolog search for *ENSCJPG00005003198* on GRCg7b genome

The sequence of *ENSCJPG00005003198* is analogous to the 27768284–27768760 MHM region on the Z chromosome of the GRCg7b genome.


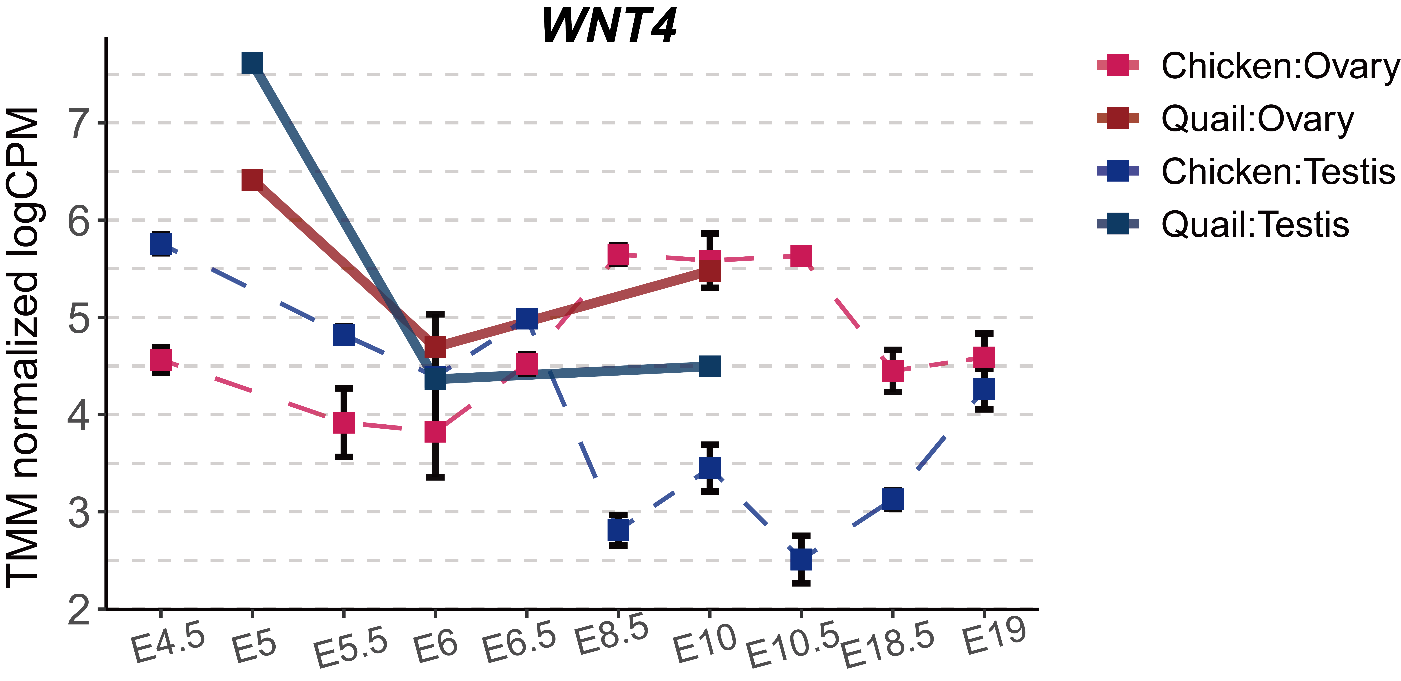


Supplementary Figure S11. The expression level of *WNT4* in the embryonic stage of quail and chicken

The X-axis represents the embryonic developmental stages of birds from E4.5 to E19, as defined by Eyal-Giladi and Kochav. The Y-axis represents the expression level of the *WNT4* gene.


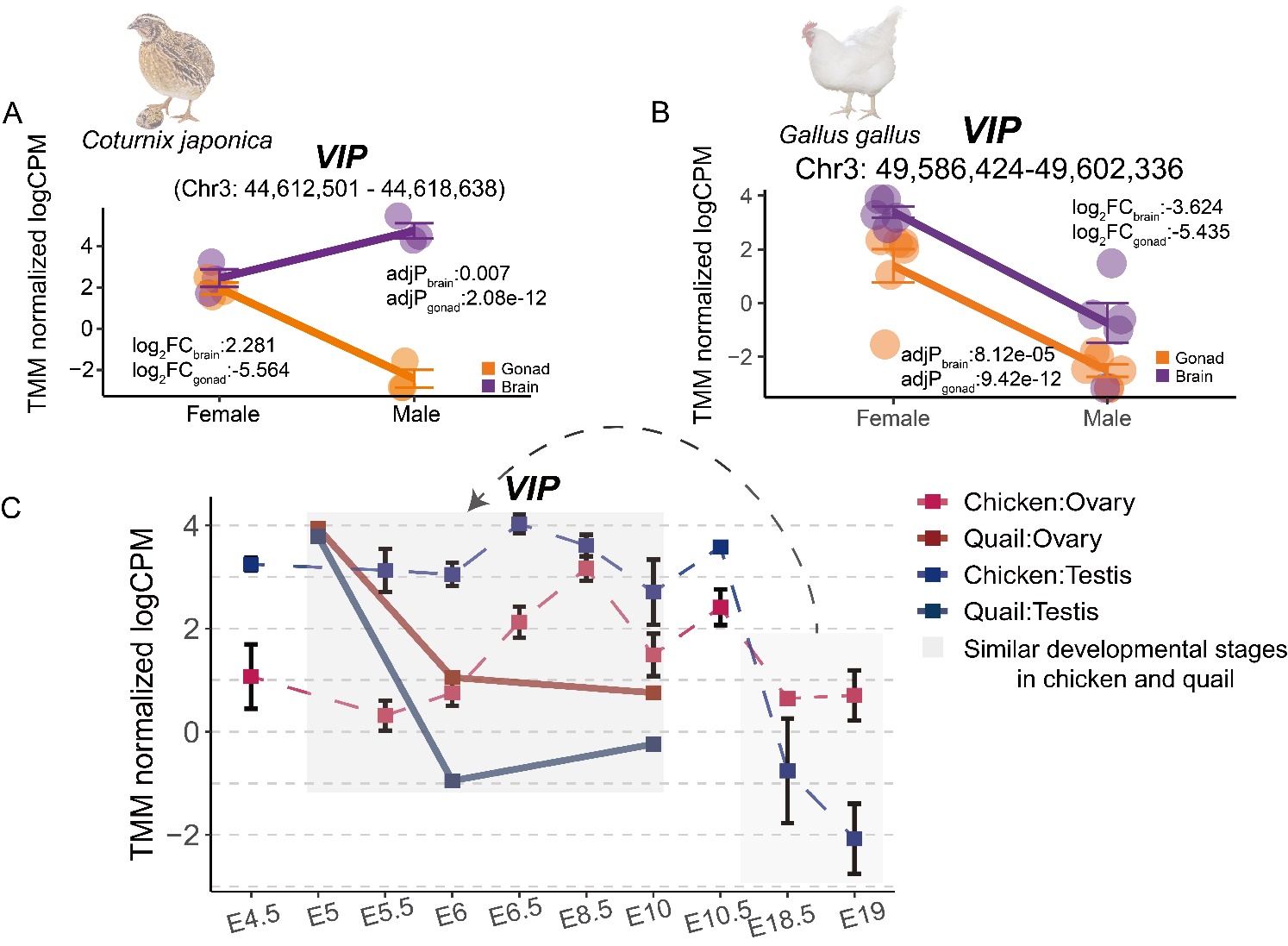


Supplementary Figure S12. The expression level of *VIP* chicken and quail

**(A)** Gene expression pattern of *VIP* in brain and reproductive organ in quail and **(B)** in chicken. **(C)** The X-axis represents the embryonic developmental stages of birds from E4.5 to E19, as defined by Eyal-Giladi and Kochav. The Y-axis represents the expression level of the *VIP* gene. Shades of gray represent similar developmental stages, presumed from the embryonic stages of chicken and quail.
